# Low serum vitamin D concentrations are associated with obese but not lean NAFLD: a cross-sectional study

**DOI:** 10.1186/s12937-021-00690-9

**Published:** 2021-04-01

**Authors:** Qinqiu Wang, Xiaoying Shi, Jinghua Wang, Juanwen Zhang, Chengfu Xu

**Affiliations:** 1 Department of Gastroenterology, the First Affiliated Hospital, Zhejiang University School of Medicine, 79 Qingchun Road, Hangzhou, 310003 China; 2 Department of Laboratory Medicine, the First Affiliated Hospital, Zhejiang University School of Medicine, Hangzhou, 310003 China

**Keywords:** Non-alcoholic fatty liver disease, Vitamin D, Obesity, Body mass index, Cross-sectional study

## Abstract

**Background:**

A low serum vitamin D concentration has been reported to be associated with an increased risk of non-alcoholic fatty liver disease (NAFLD); however, whether lean or obese individuals show a similar association between vitamin D and NAFLD remains speculative. This study aimed to explore the relationship between serum vitamin D concentration and NAFLD in lean and obese Chinese adults.

**Methods:**

This cross-sectional study included 2538 participants (1360 men and 1178 women) who underwent health checkups at the First Affiliated Hospital, Zhejiang University School of Medicine in 2019. NAFLD was diagnosed by liver ultrasound excluding other causes. The association of serum vitamin D concentration with NAFLD was analyzed in lean and obese participants.

**Results:**

The overall prevalence of NAFLD was 33.61% (13.10% in lean and 53.32% in obese) in this study population. The serum vitamin D levels of obese NAFLD patients were lower than those of obese NAFLD-free controls. However, the serum vitamin D levels of lean NAFLD patients were comparable to those of lean NAFLD-free controls. Serum vitamin D level was negatively correlated with the prevalence of NAFLD in obese but not lean participants. Serum vitamin D level was independently associated with the risk of NAFLD in obese participants, with an adjusted odds ratio (95% CI) of 0.987 (0.981–0.993). However, serum vitamin D level was not related to the risk of NAFLD in lean participants.

**Conclusions:**

A low serum vitamin D level is associated with NAFLD in obese but not lean participants.

**Supplementary Information:**

The online version contains supplementary material available at 10.1186/s12937-021-00690-9.

## Background

Non-alcoholic fatty liver disease (NAFLD) is defined as hepatic steatosis when liver lipid deposition is not secondarily caused by heavy drinking or other known etiologies with or without inflammation and fibrosis [1]. NAFLD is currently one of the world’s most common chronic liver diseases, affecting approximately 29.2% of adults in China [2]. NAFLD can lead to simple steatosis, steatohepatitis, cirrhosis, and even hepatocellular carcinoma [3, 4]. Patients with NAFLD have increased risks of cardiovascular disease, stroke, type 2 diabetes, and extrahepatic malignancies [5–8]. The high prevalence and serious clinical harms of NAFLD have made it a global research hotspot in recent years [9].

Obesity is closely related to NAFLD; the prevalence of NAFLD is higher in obese individuals than in lean individuals, and the accompanying risks are greater [10]. Recently, though, many studies have shown that non-obese people also have a high prevalence of NAFLD [11, 12]. We have previously reported that in China, the prevalence of NAFLD in the non-obese population is 7.3, and 8.9% of non-obese adults developed NAFLD during a 5-year follow-up [13]. Compared with obese NAFLD patients, lean NAFLD patients are usually asymptomatic and difficult to diagnose, but in fact, they also have severe liver histological necrotizing inflammation and high mortality [14]. NAFLD in the non-obese population may also cause significant health problems [15]. Therefore, the identification, diagnosis, and treatment of non-obese NAFLD is very important.

The risk factors for non-obese NAFLD remain unclear. Some previous cross-sectional studies showed that vitamin D deficiency was associated with an increased risk of NAFLD, and vitamin D levels were negatively associated with the severity of NAFLD [16, 17]. Several prospective studies also pointed out that serum vitamin D deficiency was accompanied by an increased risk of incident NAFLD [18–21]. Our recent study showed that serum vitamin D levels in high-fat diet-fed mice were significantly decreased, and vitamin D supplementation ameliorated high-fat diet-induced hepatic steatosis in mice [22]. Vitamin D supplementation could also improve hepatic steatosis in patients with NAFLD [23, 24]. However, it is unclear whether obesity affects the correlation between vitamin D concentration and NAFLD, and whether serum vitamin D concentration is related to NAFLD in lean individuals.

In this study, we aimed to explore the correlation between serum vitamin D concentration and NAFLD in obese and lean Chinese adults.

## Methods

### Participants

We enrolled adults who underwent health checkups at the First Affiliated Hospital, Zhejiang University School of Medicine in 2019 as participants in our cross-sectional study. The analysis included participants with complete anthropometric and biochemical data records (including serum vitamin D concentrations) and liver ultrasound results. We excluded the following participants: (i) participants with incomplete anthropometric and biochemical data; (ii) men with alcohol consumption > 210 g/week and women with alcohol consumption > 140 g/week; (iii) participants with other chronic liver diseases caused by autoimmune hepatitis or viral hepatitis; and (iv) participants who use hepatotoxic drugs (such as sulfonamide and azithromycin).

The personal information of all of the participants was anonymous. The study was approved by the Ethical Committee of the First Affiliated Hospital, Zhejiang University School of Medicine.

### Clinical examinations

Clinical examinations included questionnaires, medical history, anthropometric measurements, and biochemical analysis. During the examination, the physician recorded the medical history (including previous diseases and drug prescriptions) and drinking frequency and amount. The smoking history was also recorded and distinguished as yes or no.

The anthropometric measurements were performed as previously described, including body weight, standing height, waist circumference, and blood pressure [25, 26]. Weight and height were measured when the patient was wearing light clothing and no shoes. Waist circumference was measured after the patient exhaled, with the tape measure placed between the lowest rib and the upper edge of the iliac crest. Blood pressure was measured after resting for 5 min. Body mass index (BMI) was calculated as the weight (kg) divided by height (m) squared.

Fasting blood samples were taken from the anterior cubital vein and were used for biochemical analysis. Measurements included liver enzymes, blood lipids, glucose, and uric acid. All of the biochemical values were measured by a Hitachi 7600 clinical analyzer (Hitachi, Tokyo, Japan) using standard methods. Serum 25-hydroxyvitamin D levels were measured with the electro-chemiluminescence immunoassay (ECLIA) platform using the Roche cobas e602 analyzer (Roche Diagnostics GmbH, Germany).

### Diagnostic criteria and definitions

Lean was defined as BMI < 24 kg/m^2^, and obesity was defined as BMI ≥ 24 kg/m^2^ [27]. The quartiles of serum vitamin D levels were defined as follows: quartile 1, vitamin D <  45.5 nmol/L; quartile 2, 45.5–59.5 nmol/L; quartile 3, 59.6–74.2 nmol/L; and quartile 4, vitamin D ≥ 74.3 nmol/L. Vitamin D deficiency was defined as vitamin D <  50.0 nmol/L; vitamin D insufficiency as 50.0–74.9 nmol/L; and vitamin D sufficiency as vitamin D ≥ 75.0 nmol/L [28] . Central obesity was defined as waist circumference ≥ 90 cm in males and ≥ 80 cm in females according to the International Diabetes Federation cut-offs for Chinese adults [29].

### Diagnosis of NAFLD

An abdominal ultrasound examination was performed by experienced ultrasonographers using the Toshiba Nemio 20 ultrasound system (Toshiba, Tokyo, Japan) with a 3.5 MHz probe. The ultrasonographers were unaware of the study’s purpose and laboratory values. Fatty liver disease was diagnosed according to the standards of the Chinese Liver Disease Association, wherein the diagnosis requires the presence of significant hepatic steatosis confirmed by imaging or histological examination, in the absence of other causes for fatty liver diseases such as overconsumption of alcohol [30].

### Statistical analysis

The statistical analysis was performed by SPSS (SPSS, Chicago, IL) for Mac version 18.0. Continuous variables were presented as mean ± SD or median and interquartile range (IQR). Student’s *t* test was applied to compare continuous data, and the *χ*^2^ test was applied to compare categorical variables. The Cochran-Armitage trend test showed the trend of prevalence. Logistic regression analysis was used to identify possible risk factors for NAFLD. *P* <  0.05 (two-tailed test) was considered to be statistically significant.

## Results

### Clinical characteristics of the study population

A total of 2538 participants (1360 men and 1178 women) were included in this study, and 853 (33.61%) had NAFLD. The prevalence of NAFLD was 13.10% in lean participants (BMI < 24 kg/m^2^) and 53.32% in obese participants (BMI ≥ 24 kg/m^2^). Comparing clinical characteristics based on NAFLD status (Table 1), we found that both lean and obese NAFLD patients had higher BMI, larger waist circumference, higher systolic and diastolic blood pressure, and elevated serum levels of alanine aminotransferase, γ-glutamyl transpeptidase, triglyceride, uric acid, and fasting glucose, but lower serum HDL-cholesterol levels than corresponding controls. In addition, the vitamin D levels of obese NAFLD patients were lower than those of obese NAFLD-free controls (59.03 ± 19.46 nmol/L versus 63.56 ± 22.09 nmol/L, *P* <  0.001), but this was not observed in lean participants (Table 1).

**Table 1:** Clinical characteristics of the study population according to obese and NAFLD categories

Variables	Overall (*n* = 2538)	Lean participants			Obese participants		
		NAFLD (*n* = 163)	Without NAFLD (*n* = 1081)	*P* value	NAFLD (*n* = 690)	Without NAFLD (*n* = 604)	*P* value
Age (year)	54.08 (6.85)	54.84 (6.59)	53.81 (6.96)	0.078	54.23 (6.60)	54.18 (6.99)	0.901
Male gender (%)	1360 (53.39)	86 (52.76)	430 (39.78)	0.002	485 (70.29)	359 (59.44)	< 0.001
Body mass index (kg/m^2^)	24.22 (2.96)	22.77 (1.14)	21.71 (1.54)	< 0.001	27.09 (2.18)	25.83 (1.73)	< 0.001
Waist circumference (cm)	85.13 (8.67)	83.34 (5.12)	78.66 (6.12)	< 0.001	92.88 (6.39)	88.35 (6.37)	< 0.001
Systolic blood pressure (mmHg)	127.93 (18.38)	127.64 (16.86)	122.77 (18.51)	0.002	134.37 (17.41)	129.88 (16.89)	< 0.001
Diastolic blood pressure (mmHg)	78.37 (11.56)	78.08 (9.98)	74.77 (11.26)	< 0.001	82.96 (10.72)	79.65 (11.34)	< 0.001
Alanine aminotransferase (U/L)	23.21 (19.06)	28.64 (45.74)	18.24 (12.69)	0.004	30.78 (16.61)	22.00 (15.60)	< 0.001
γ-Glutamyl Transpeptidase (U/L)	29.90 (31.84)	35.50 (35.39)	23.00 (31.34)	< 0.001	40.49 (32.64)	28.64 (26.98)	< 0.001
Triglyceride (mmol/L)	1.63 (1.14)	2.02 (1.16)	1.26 (0.82)	< 0.001	2.17 (1.43)	1.54 (0.95)	< 0.001
HDL-cholesterol (mmol/L)	1.22 (0.34)	1.12 (0.26)	1.36 (0.36)	< 0.001	1.04 (0.25)	1.19 (0.28)	< 0.001
LDL-cholesterol (mmol/L)	2.75 (0.72)	2.83 (0.79)	2.69 (0.68)	0.016	2.79 (0.75)	2.79 (0.74)	0.839
Serum uric acid (μmol/L)	325.71 (81.72)	332.94 (73.72)	293.72 (71.12)	< 0.001	369.41 (78.22)	331.10 (80.78)	< 0.001
Fasting blood glucose (mmol/L)	5.21 (1.21)	5.53 (1.34)	4.99 (1.03)	< 0.001	5.57 (1.50)	5.12 (0.94)	< 0.001
Vitamin D (nmol/L)	61.21 (21.64)	60.95 (18.54)	61.32 (22.97)	0.844	59.03 (19.46)	63.56 (22.09)	< 0.001

Data are expressed as mean (SD)

*HDL-C* high-density lipoprotein cholesterol, *LDL-C* low-density lipoprotein cholesterol, *NAFLD* nonalcoholic fatty liver disease

### Association of serum vitamin D level with the prevalence of NAFLD

We classified all of the participants into quartiles by their serum vitamin D levels and analyzed the association of vitamin D quartiles with the prevalence of NAFLD. We found that serum vitamin D quartiles were negatively associated with the prevalence of NAFLD in obese participants (Table 2). The prevalence of NAFLD was 56.54, 59.77, 52.15, and 44.51% in the first, second, third, and fourth quartiles of serum vitamin D in obese participants, respectively (*P* for trend < 0.01; Table 2). However, serum vitamin D quartiles were not associated with the prevalence of NAFLD in lean participants (Table 2).

**Table 2:** Association of vitamin D quartiles with prevalence of NAFLD in lean and obese participants

VD quartiles	Lean participants						Obese participants					
	Total	NAFLD	PR%	PR	χ^2^	*P* value	Total	NAFLD	PR%	PR	χ^2^	*P* value
Quartile 1	327	37	11.31	0.95			306	173	56.54	1.27		
Quartile 2	292	38	13.01	1.09			343	205	59.77	1.34		
Quartile 3	306	50	16.34	1.37			326	170	52.15	1.17		
Quartile 4	319	38	11.91	1.00	4.133	0.247	319	142	44.51	1.00	17.118	< 0.01

Participants were classified into quartiles according to their serum vitamin D levels: quartile 1, < 45.5 nmol/L; quartile 2, 45.5–59.5 nmol/L; quartile 3, 59.6–74.2 nmol/L; and quartile 4, ≥ 74.3 nmol/L

*VD* vitamin D, *PR%* prevalence rate, *PR* prevalence ratio

We also divided all of the participants into three groups according to their vitamin D adequacy status and analyzed the association of vitamin D adequacy status with the prevalence of NAFLD in lean and obese participants. In the obese group, we found that participants with vitamin D deficiency had the highest prevalence of NAFLD (57.60%), followed by those with vitamin D insufficiency (55.73%), and then those with vitamin D sufficiency (43.23%) (*P* for trend < 0.001; Table 3). In the lean group, the prevalence of NAFLD was comparable among participants with vitamin D deficiency and those with vitamin D sufficiency (11.14% versus 10.89%, Table 3).

**Table 3:** Association of vitamin D sufficiency with prevalence of NAFLD in lean and obese participants

VD classification	Lean participants						Obese participants					
	Total	NAFLD	PR%	PR	χ^2^	*P*-value	Total	NAFLD	PR%	PR	χ^2^	*P* value
VD deficiency	422	47	11.14	1.02			408	235	57.60	1.33		
VD insufficiency	519	83	15.99	1.47			576	321	55.73	1.29		
VD sufficiency	303	33	10.89	1.00	6.539	0.038	310	134	43.23	1.00	17.034	< 0.001

*VD* vitamine D, *PR%* prevalence rate, *PR* prevalence ratio

Participants were classified into three groups according to their serum VD levels: VD deficiency, < 50.0 nmol/L; VD insufficiency, 50.0–74.9 nmol/L; and VD sufficiency, ≥ 75.0 nmol/L.

### Association of serum vitamin D level with risk of NAFLD

We performed multiple logistic regression analyses to explore the risk factors of NAFLD in lean and obese participants. We found that male sex, high BMI and waist circumference, high serum levels of alanine aminotransferase, triglyceride, LDL-cholesterol, uric acid, and fasting blood glucose, and low serum levels of HDL-cholesterol were correlated with increased risks of NAFLD in both lean and obese participants (Table 4). We also found that serum vitamin D concentration was a protection factor associated with the risk of NAFLD in obese participants, with an adjusted odds ratio (OR) (95% CI) of 0.987 (0.981–0.993). However, serum vitamin D concentration was not associated with the risk of NAFLD in lean participants (Table 4).

**Table 4:** Logistic regression analysis for factors associated with risk of NAFLD in lean and obese participants

Variables	Lean participants			Obese participants		
	Wald *χ*^2^	OR (95% CI)	*P* value	Wald *χ*^2^	OR (95% CI)	*P* value
Male gender	10.789	2.234 (1.383–3.608)	0.001	5.315	1.494 (1.062–2.101)	0.021
Body mass index (kg/m^2^)	13.163	1.439 (1.182–1.751)	< 0.001	13.794	1.192 (1.086–1.307)	< 0.001
Waist circumference (cm)	9.856	1.074 (1.027–1.122)	0.002	11.344	1.052 (1.021–1.084)	0.001
Alanine aminotransferase (U/L)	11.112	1.019 (1.008–1.030)	0.001	27.934	1.026 (1.016–1.036)	< 0.001
Triglyceride (mmol/L)	15.537	1.460 (1.210–1.762)	< 0.001	12.495	1.302 (1.125–1.507)	< 0.001
HDL-cholesterol (mmol/L)	11.076	0.271 (0.125–0.584)	0.001	19.940	0.271 (0.153–0.480)	< 0.001
LDL-cholesterol (mmol/L)	8.583	1.469 (1.136–1.899)	0.003	4.322	1.205 (1.011–1.437)	0.038
Serum uric acid (μmol/L)	9.342	1.005 (1.002–1.008)	0.002	13.417	1.004 (1.002–1.006)	< 0.001
Fasting blood glucose (mmol/L)	11.160	1.251 (1.097–1.427)	0.001	15.639	1.274 (1.130–1.436)	< 0.001
Vitamin D (nmol/L)	–	–	–	18.017	0.987 (0.981–0.993)	< 0.001

*OR* odds ratio, *CI* confidence interval, *HDL-cholesterol* high-density lipoprotein cholesterol, *LDL-C* low-density lipoprotein cholesterol

We divided all of the participants into the categories of central obesity and non-central obesity according to their waist circumference. We found that, in the central obesity group, vitamin D concentration was a protective factor for NAFLD with an adjusted OR (95% CI) of 0.994 (0.989–0.999), but this was not the case in the non-central obesity group (Supplementary Table S1). Moreover, we found that regardless of metabolic syndrome status, serum vitamin D concentration was a protective factor for NAFLD in obese participants but not in lean participants (Supplementary Table S2).

We also analyzed the correlation between vitamin D quartiles and the risk of NAFLD (Table 5). Among obese participants, compared with participants in the fourth quartile, participants with serum vitamin D levels in the first, second, and third quartiles all showed increased risk of NAFLD, with the adjusted OR (95% CI) of 1.973 (1.359–2.865), 1.964 (1.371–2.815), and 1.420 (0.992–2.033), respectively. However, the risk of NAFLD was comparable among lean participants with serum vitamin D levels in all of the quartiles (Table 5). Similar results were observed by dividing all of the participants into central obesity and non-central obesity categories according to their waist circumference (Supplementary Table S3).

**Table 5:** Association of serum vitamin D quartiles with risk of NAFLD in lean and obese participants

Lean	Models	Odds ratios (95% confidence interval)				*χ*^2^ value	*P* value
		Quartile 1 (*n* = 327)	Quartile 2 (*n* = 292)	Quartile 3 (*n* = 306)	Quartile 4 (*n* = 319)		
	Model 1	0.943 (0.583–1.527)	1.106 (0.684–1.789)	1.444 (0.917–2.275)	1	4.100	0.251
	Model 2	1.248 (0.742–2.099)	1.334 (0.800–2.225)	1.557 (0.961–2.522)	1	3.305	0.347
	Model 3	1.176 (0.673–2.055)	1.063 (0.609–1.853)	1.535 (0.916–2.571)	1	3.238	0.356
Obese	Models	Odds ratios (95% confidence interval)				*χ*^2^ value	*P* value
		Quartile 1 (*n* = 306)	Quartile 2 (*n* = 343)	Quartile 3 (*n* = 326)	Quartile 4 (*n* = 319)		
	Model 1	1.621 (1.182–2.224)	1.852 (1.360–2.521)	1.358 (0.996–1.852)	1	16.980	0.001
	Model 2	1.941 (1.368–2.753)	1.963 (1.404–2.746)	1.508 (1.080–2.105)	1	19.461	< 0.001
	Model 3	1.973 (1.359–2.865)	1.964 (1.371–2.815)	1.420 (0.992–2.033)	1	17.747	< 0.001

Model 1 was unadjusted

Model 2 was adjusted for age, gender, waist circumference and body mass index

Model 3 was further adjusted for systolic and diastolic blood pressure, alanine aminotransferase, γ-glutamyl transpeptidase, triglyceride, HDL-cholesterol, LDL-cholesterol, fasting blood glucose and serum uric acid

Participants were classified into quartiles according to their serum vitamin D levels: quartile 1, < 45.5 nmol/L; quartile 2, 45.5–59.5 nmol/L; quartile 3, 59.6–74.2 nmol/L; and quartile 4, ≥ 74.3 nmol/L

We further analyzed the correlation between vitamin D adequacy status and risk of NAFLD (Table 6). Among obese participants, those with vitamin D deficiency showed an increased risk of NAFLD compared with those with vitamin D sufficiency, with an adjusted OR (95% CI) of 2.076 (1.462–2.950). However, among lean participants, vitamin D deficiency was not associated with risk of NAFLD in adjusted models (Table 6). These results showed that decreased serum vitamin D concentration was associated with an increased risk of NAFLD in obese but not lean participants.

**Table 6:** Association of vitamin D sufficiency status with risk of NAFLD in lean and obese participants

Lean	Models	Odds ratios (95% confidence interval)			*χ*^2^ value	*P* value
		VD deficiency (*n* = 422)	VD insufficiency (*n* = 519)	VD sufficiency (*n* = 303)		
	Model 1	1.025 (0.640–1.644)	1.558 (1.012–2.397)	1	6.474	0.039
	Model 2	1.335 (0.803–2.221)	1.726 (1.091–2.730)	1	5.736	0.057
	Model 3	1.192 (0.693–2.050)	1.602 (0.982–2.612)	1	4.145	0.126
Obese	Models	Odds ratios (95% confidence interval)			*χ*^2^ value	*P* value
		VD deficiency (*n* = 408)	VD insufficiency (*n* = 576)	VD sufficiency (*n* = 310)		
	Model 1	1.784 (1.324–2.405)	1.653 (1.252–2.184)	1	16.853	< 0.001
	Model 2	2.055 (1.479–2.854)	1.792 (1.325–2.423)	1	20.578	< 0.001
	Model 3	2.076 (1.462–2.950)	1.730 (1.252–2.390)	1	17.737	< 0.001

Model 1 was unadjusted

Model 2 was adjusted for age, gender, waist circumference and body mass index

Model 3 was further adjusted for systolic and diastolic blood pressure, alanine aminotransferase, γ-glutamyl transpeptidase, triglyceride, HDL-cholesterol, LDL-cholesterol, fasting blood glucose and serum uric acid

Participants were classified into three groups according to their serum VD levels: VD deficiency, < 50.0 nmol/L; VD insufficiency, 50–74.9 nmol/L; and VD sufficiency, ≥ 75.0 nmol/L.

## Discussion

In this study, we explored the correlation between serum vitamin D concentration and NAFLD in Chinese adults. We found that serum vitamin D concentrations of obese NAFLD patients were lower than those of obese controls without NAFLD. We also found that serum vitamin D concentration was negatively correlated with the prevalence of NAFLD in obese but not lean participants. Our further analysis showed that decreased serum vitamin D concentration and vitamin D deficiency were associated with an increased risk of NAFLD in obese but not lean participants. These findings suggested a significant correlation between serum vitamin D concentration and NAFLD in obese but not lean participants.

Several studies have reported that there is a significant correlation between serum vitamin D concentration and NAFLD [31, 32], and this was confirmed by a meta-analysis including 12,794 participants of 17 studies [33]. Moreover, low serum vitamin D concentration is related to greater severity of hepatic steatosis and necrotizing inflammation in both children and adults [34, 35]. Preclinical investigations found that vitamin D supplementation significantly improved liver steatosis in high-fat diet-fed mice [22]. In addition, both our research team and others found that vitamin D receptor (VDR) is upregulated in the steatotic liver and may be a therapeutic target for NAFLD [22, 36]. As we know, low serum vitamin D levels are more commonly observed in obese than in lean individuals [37]. However, whether lean and obese individuals show a similar association of vitamin D with NAFLD remains speculative and should be investigated. In this study, we provided evidence that low serum vitamin D levels are associated with obese but not lean populations.

The explanation for why obese and lean individuals have inconsistent correlations between vitamin D and NAFLD remains unclear, although several possibilities exist. First, obesity could be a contributing factor to low vitamin D levels [38, 39]. With fewer outdoor activities and thus less exposure to sunlight, obese individuals may have decreased vitamin D synthesized in the liver or percutaneously [8]. A genetic study showed that each increase in BMI will reduce serum vitamin D concentration by 1.15% [40]. Second, patients with vitamin D deficiency have higher serum levels of proinflammatory cytokines, which promote the development of NAFLD [41]. In the non-alcoholic steatohepatitis (NASH) stage, vitamin D deficiency can also actively regulate the synthesis of endogenous fatty acids in the liver by weakening enterohepatic circulation [42]. Third, vitamin D regulates lipid transport by binding with vitamin D receptor and activating Hepatocyte nuclear factor 4α (HNF4α) [22]. Therefore, under vitamin D deficiency conditions, the flow of free fatty acids (FFAs) in the blood increases, and fat deposition into hepatocytes is accelerated, contributing to the progress of NAFLD [43]. Further research is needed to clarify these possibilities.

Recently, researchers have subdivided NAFLD into obese and lean subtypes, and many studies have focused on lean NAFLD [44–46]. Vitamin D concentration is closely related to NAFLD, and vitamin D deficiency is considered a risk factor for NAFLD [32, 43]. However, it was not clear whether vitamin D concentration was also associated with lean NAFLD. In this study, we found that low vitamin D concentration is associated with obese but not lean NAFLD. Based on the cross-sectional study, we hypothesized that the vitamin D concentration may be an important predictor of NAFLD in the obese but not lean population.

Some limitations of this study should be acknowledged. First, our NAFLD was diagnosed based on ultrasound. Although ultrasound NAFLD diagnosis has been widely used clinically as a screening method for hepatic steatosis, it is still insufficient to detect mild steatosis, and it cannot replace the gold standard for liver biopsy. The correlation between vitamin D levels and NAFLD histological severity was not explored in this study. Second, this is a single-center cross-sectional study. Our sample size may be insufficient to represent the entire Chinese adult population, and further multi-center cohort studies are needed. Third, the BMI classification criteria applied in this study are limited to East Asian people, so our findings may not be generalizable to other ethnic populations. Fourth, our study is a cross-sectional study, and further prospective studies are needed to analyze the causal relationship between vitamin D deficiency and the onset of NAFLD.

## Conclusions

Our cross-sectional study provides evidence that there is a significant correlation between serum vitamin D concentration and NAFLD in obese but not lean participants. Further research is needed to explore the complicated relationships and possible mechanisms between obesity, vitamin D level, and NAFLD.

## Supplementary Information

**Additional file 1: Supplementary Table S1**. Logistical regression analysis for factors with risk of NAFLD in lean and central obese participants

**Additional file 2: Supplementary Table S2**. Association of serum vitamin D concentrations with risk of NAFLD in lean and obese participants with or without metabolic syndrome

**Additional file 3: Supplementary Table S3**. Association of serum vitamin D quartiles with risk of NAFLD in lean and central obese participants

## Data Availability

The data that support the findings of this study are available from the First Affiliated Hospital, Zhejiang University School of Medicine but restrictions apply to the availability of these data, which were used under license for the current study, and so are not publicly available. Data are however available from the authors upon reasonable request and with permission of the First Affiliated Hospital, Zhejiang University School of Medicine.

## Abbreviations

NAFLD: Non-alcoholic fatty liver disease
BMI: Body mass index
ECLIA: Electro-chemiluminescence immunoassay
IQR: Interquartile range
VDR: Vitamin D receptor
NASH: Non-alcoholic steatohepatitis
PPAR-γ: Peroxisome proliferator-activated receptor γ
FFA: Free fatty acid
