# Supplementary material for: Low serum vitamin D concentrations are associated with obese but not lean NAFLD: a cross-sectional study
Source: Nutr J. 2021 Apr 1;20:30. doi: 10.1186/s12937-021-00690-9 (PMC8017627; doi:10.1186/s12937-021-00690-9)
Supplement: Supplementary file 1 — Additional file 1: Supplementary Table S1. Logistical regression analysis for factors with risk of NAFLD in lean and central obese participants [file 12937_2021_690_MOESM1_ESM.docx]

**Supplementary Table S1. Logistical regression analysis for factors with risk of NAFLD in lean and central obese participants**

| Variables | Lean participants | | |  | Central obese participants | | | |
| --- | --- | --- | --- | --- | --- | --- | --- | --- |
|  | Wald χ^2^ | OR (95% CI) | *P* value |  | Wald χ^2^ | OR (95% CI) | *P* value |  |
| Body mass index (kg/m^2^) | 117.396 | 1.617 (1.483–1.764) | <0.001 |  | 165.042 | 1.437 (1.359–1.518) | <0.001 |  |
| Alanine aminotransferase (U/L) | 39.398 | 1.036 (1.025–1.048) | <0.001 |  | 96.989 | 1.053 (1.042–1.063) | <0.001 |  |
| γ-Glutamyl transpeptidase (U/L) | 10.249 | 0.922 (0.877–0.969) | 0.001 |  | 63.684 | 1.023 (1.018–1.029) | <0.001 |  |
| Triglycerides (mmol/L) | 72.165 | 1.897 (1.636–2.198) | <0.001 |  | 96.235 | 2.176 (1.863–2.542) | <0.001 |  |
| HDL-cholesterol (mmol/L) | 71.434 | 0.099 (0.058–0.170) | <0.001 |  | 131.379 | 0.065 (0.041–0.104) | <0.001 |  |
| LDL-cholesterol (mmol/L) | 63.267 | 4.269 (2.985–6.104) | <0.001 |  | 60.265 | 3.929 (2.781–5.550) | <0.001 |  |
| Serum uric acid (μmol/L) | 82.046 | 1.009 (1.007–1.011) | <0.001 |  | 117.565 | 1.009 (1.007–1.010) | <0.001 |  |
| Fasting blood glucose (mmol/L) | 20.323 | 1.308 (1.164–1.471) | <0.001 |  | 44.616 | 1.529 (1.350–1.732) | <0.001 |  |
| Vitamin D (nmol/L) | - | - | - |  | 4.898 | 0.994 (0.989–0.999) | 0.027 |  |

Central obesity was defined as waist circumference  ≥ 90 cm in males and  ≥ 80 cm in females according to International Diabetes Federation cut offs for Chinese.

OR, odds ratio; CI, confidence interval; HDL-cholesterol, high-density lipoprotein cholesterol; LDL-cholesterol, low-density lipoprotein cholesterol.
