# Supplementary material for: Low serum vitamin D concentrations are associated with obese but not lean NAFLD: a cross-sectional study
Source: Nutr J. 2021 Apr 1;20:30. doi: 10.1186/s12937-021-00690-9 (PMC8017627; doi:10.1186/s12937-021-00690-9)
Supplement: Supplementary file 2 — Additional file 2: Supplementary Table S2. Association of serum vitamin D concentrations with risk of NAFLD in lean and obese participants with or without metabolic syndrome [file 12937_2021_690_MOESM2_ESM.docx]

**Supplementary Table S2. Association of serum vitamin D concentrations with risk of NAFLD in lean and obese participants with or without metabolic syndrome**

| With/without MS | Lean participants | | |  | Obese participants | | | |
| --- | --- | --- | --- | --- | --- | --- | --- | --- |
|  | Wald χ^2^ | OR (95% CI) | *P* value |  | Wald χ^2^ | OR (95% CI) | *P* value |  |
| With MS | 3.144 | 0.976 (0.950–1.003) | 0.076 |  | 6.055 | 0.985 (0.974–0.997) | 0.014 |  |
| Without MS | 0.122 | 0.998 (0.988–1.008) | 0.727 |  | 11.908 | 0.987 (0.979–0.994) | 0.001 |  |

The ORs were adjusted for age, gender, waist circumference, body mass index, systolic and diastolic blood pressure, alanine aminotransferase, γ-glutamyl transpeptidase, triglyceride, HDL-cholesterol, LDL-cholesterol, fasting blood glucose and serum uric acid.

OR, odds ratio; CI, confidence interval.
