# Supplementary material for: Low serum vitamin D concentrations are associated with obese but not lean NAFLD: a cross-sectional study
Source: Nutr J. 2021 Apr 1;20:30. doi: 10.1186/s12937-021-00690-9 (PMC8017627; doi:10.1186/s12937-021-00690-9)
Supplement: Supplementary file 3 — Additional file 3: Supplementary Table S3. Association of serum vitamin D quartiles with risk of NAFLD in lean and central obese participants [file 12937_2021_690_MOESM3_ESM.docx]

**Supplementary Table S3. Association of serum vitamin D quartiles with risk of NAFLD in lean and central obese participants**

| Lean | Models | Odds ratios (95% confidence interval) | | | | *χ*^2^ value | *P* value |
| --- | --- | --- | --- | --- | --- | --- | --- |
|  |  | Q1 (*n*=324) | Q2 (*n*=298) | Q3 (*n*=327) | Q4 (*n*=340) |  |  |
|  | Model 1 | 1.225 (0.794–1.889) | 1.418 (0.921–2.184) | 1.658 (1.096–2.508) | 1 | 6.203 | 0.102 |
|  | Model 2 | 1.592 (0.984–2.576) | 1.567 (0.980–2.506) | 1.608 (1.028–2.513) | 1 | 5.513 | 0.138 |
|  | Model 3 | 1.782 (1.067–2.979) | 1.425 (0.862–2.687) | 1.660 (1.026–2.687) | 1 | 5.924 | 0.115 |
| Central obese | Models | Odds ratios (95% confidence interval) | | | | *χ*^2^ value | *P* value |
|  |  | Q1 (*n*=309) | Q2 (*n*=337) | Q3 (*n*=305) | Q4 (*n*=298) |  |  |
|  | Model 1 | 1.280 (0.930–1.761) | 1.561 (1.140–2.136) | 1.231 (0.894–1.696) | 1 | 7.797 | 0.050 |
|  | Model 2 | 1.697 (1.179–2.641) | 1.853 (1.301–2.641) | 1.506 (1.054–2.152) | 1 | 13.185 | 0.004 |
|  | Model 3 | 1.606 (1.088–2.370) | 1.761 (1.201–2.581) | 1.365 (0.930–2.004) | 1 | 9.470 | 0.024 |

Central obesity was defined as waist circumference  ≥ 90 cm in males and  ≥ 80 cm in females according to International Diabetes Federation cut-offs for Chinese.

Model 1 was unadjusted.

Model 2 was adjusted for age, gender, waist circumference and body mass index.

Model 3 was further adjusted for systolic and diastolic blood pressure, alanine aminotransferase, γ-glutamyl transpeptidase, triglyceride, HDL-cholesterol, LDL-cholesterol, fasting blood glucose and serum uric acid.

Participants were classified into quartiles according to their serum vitamin D levels: quartile 1, < 45.5 nmol/L; quartile 2, 45.5-59.5 nmol/L; quartile 3, 59.6-74.2 nmol/L; and quartile 4, ≥ 74.3 nmol/L.
